# Gender-Specific Wage Structure and the Gender Wage Gap in the U.S. Labor Market

**DOI:** 10.1007/s11205-022-03030-4

**Published:** 2022-11-21

**Authors:** Assaf Rotman, Hadas Mandel

**Affiliations:** Department of Sociology and Anthropology, Tel Aviv University, 69978 Tel Aviv, Israel

**Keywords:** Gender pay gap, Gender inequality, Wage structure, Returns to skills, Returns to education, Returns to work experience

## Abstract

This paper challenges the predominant conceptualization of the wage structure as gender-neutral, emphasizing the contribution that this makes to the gender wage gap. Unlike most decomposition analyses, which concentrated on gender differences in productivity-enhancing characteristics (the ‘explained’ portion), we concentrate on the ‘wage structure’ (the ‘unexplained’ portion), which can be defined as the market returns to productivity-enhancing characteristics. These returns are commonly considered a reflection of non-gendered economic forces of supply and demand, and gender differences in these returns are attributed to market failure or measurement error. Using PSID data on working-age employees from 1980 to 2010, we examine gender differences in returns to education and work experience in the U.S. labor market. Based on a threefold decomposition, we estimate the contribution of these differences to the overall pay gap. The results show that men’s returns to education and work experience are higher than women’s; and that in contrast to the well-documented trend of narrowing gender gaps in skills and earnings, the gaps in returns increase over time in men’s favor. Furthermore, the existing gender differences in returns to skills explain a much larger proportion of the gender wage gap than differences in levels of education and experience between men and women. The paper discusses the mechanisms underlying these findings.

## Introduction

The gender wage gap is the subject of an extensive and rich body of literature. Various theoretical arguments, backed by empirical evidence, have been presented over time to explain the sources of the gender wage gap, and its changes over time. A central strand of this literature concentrates on estimating the contribution of different factors to the overall gender wage gap. Studies in this line of research decompose the gender gap to its constituent components, while making a distinction between the ‘explained’ and the ‘unexplained’ portions of the gender pay gap (Kunze, 2008). The ‘explained’ portion refers to the part of the gap that is the consequence of differences between men and women in their productivity-related characteristics, such as work experience and education. The ‘unexplained’ portion is the ‘residual’, commonly presented as a rough estimate of labor market discrimination. While the ‘explained’ component of the gap has been meticulously scrutinized, the ‘unexplained’ portion is usually treated as a monolithic element, and as such is rarely given close and detailed examination despite its increasing relative size over time.

In this study, we focus on the ‘unexplained’ portion of the gender wage gap, a focus that carries theoretical and empirical contributions. Empirically, we do not leave the ‘unexplained’ portion of the wage gap as a solid irreducible block; rather, we disaggregate it to its two main components, education and work experience. This approach is uncommon in the field. The extensive literature on the gender wage gap focuses almost entirely on the ‘explained’ portion of the gap in examining gender differences in education and experience—namely differences at the individual level. Studies that decompose the gender wage gap document the importance of such differences at the individual level—especially the convergence between men and women with regard to education and experience—in explaining the decline in the gender pay gap over time (Blau & Kahn, 1997, 2017a; Kunze, 2017). The structural aspects of the gender wage gap, on the other hand, are often left unnoticed.

Theoretically, we stress that the ‘unexplained’ portion is gender specific, in contrast to the bulk of the existing research, which conceptualizes it as gender-neutral. The ‘unexplained’ portion of the wage gap relates to structural aspects of gender inequality which operate beyond the individual level, and therefore are harder to identify. This structural portion refers to the market price for productivity-enhancing skills, arguably determined by supply and demand and thus generally assumed to be gender-neutral (Blau & Kahn, 1996, 1997). Following this assumption, if the market price is not identical for men and women, it is assumed to be due to market failure (i.e., discrimination) or measurement error and misspecification. In contrast to this, the present study is motivated by the notion that structural aspects are gender-specific rather than gender-neutral; accordingly, our goal is to demonstrate how men and women are rewarded differently for their skills.

In this paper, then, we focus on the ‘unexplained’ portion of the gender wage gap, i.e., the market monetary rewards to skills, and its changing size between 1980 and 2010. Specifically, we examine how market rewards to the two prime human-capital determinants of wages—education and work experience—differ for men and women, and the extent to which these differences contribute to the gender pay gap. Since gender differences in productivity-enhancing skills have dramatically changed over the last few decades, alongside changes to the returns to these skills, we also examine how the ‘unexplained’ portion of the wage gap changes over time.

Our findings show that market rewards to education and work experience differ significantly for men and women, and that these differences account for a much larger proportion of the wage gap than differences in the levels of education and work experience between men and women. Furthermore, not only is it that this ‘explained’ portion of the gender gap smaller; its significance is in decline (see also Blau & Kahn, 2017a, 2017b), while structural aspects (the ‘unexplained’ portion) remain a powerful force behind the persistence of the gender wage gap. The durable importance of the ‘unexplained’ portion of the gender pay gap means that further improvements in women’s human capital will hardly contribute to minimizing their wage disadvantage, as their inferior wage is mainly the result of the lower evaluation of their skills. The conclusions that stem from these findings are rather clear, and should be echoed to both researchers and policy makers: women’s investment in human capital will only contribute to minimizing their economic inferiority when the ‘wage structure’—the criteria underpinning the market prices for skills and occupations—is equal for men and women. For as long as the market continues to reward men and women with equivalent skills differently, the gender gap will persist.

The paper is structured as follows: Sect. 2 critically reviews the common conceptualization of the wage structure, Sect. 3 discusses methods of decomposition of the gender wage gap and their ability to identify the contribution of the wage structure to the pay gap, Sect. 4 presents the data and variables used in the analysis, Sect. 5 presents the findings, and finally, Sect. 6 draws the conclusions and proposes the mechanisms underlying the results.

## Individual and Structural Aspects of the Gender Wage Gap

The literature explaining earnings inequality between male and female workers relies heavily on Becker’s human capital theory (Becker, 1962) and Mincer’s earnings function (Mincer, 1974) that serve as the cornerstone for understanding wage differentials in modern labor markets. In a nutshell, the human capital theory suggests that economic rewards are primarily based on ‘productivity’, and hence workers with higher productivity-enhancing characteristics will earn higher wages. Education and work experience consist the two main components of human capital, and thus have been studied extensively.

Applying this model for studying wage differences between men and women means that, theoretically, the gender wage gap (or at least a significant part of it) is a reflection of the fact that men and women entering the labor force differ in their human capital. In other words: if men generally have higher productive skills than women, this will result in wage disparities in their favor. In case of a convergence between men and women in human capital, a similar convergence in the gender wage gap should be expected as well. According to this logic, studies that decompose the gender wage gap term the portion of the gap attributed to differences in human capital between men and women the ‘explained’ portion. However, differences in human capital and other productivity-related characteristics do not explain the entire gap. The gender wage gap is also an outcome of the market price for human capital, a price that is affected by supply and demand. The market price for skills constitutes the ‘*wage structure*’, or, in other words, what employers are willing to pay for certain productivity-enhancing qualities (Blau & Kahn, 1996, 1997).

In theory, the wage structure—as the ‘real’ market value for productivity-related skills—is gender-neutral, determined by economic forces in a given market regardless of the worker’s identity. Following this assumption, if gender differences in the rewards for skills do exist it is due to market failure; therefore, these differences are conceived as the ‘unexplained’ portion of the gender wage gap. Indeed, the ‘unexplained’ portion is often presented as an estimate of ‘market discrimination’, which in decomposition reasearch relates to unequal pay for workers of different genders with the same productivity-related skills, i.e., based on their gender rather than their productivity.

Although the wage structure is assumed to be gender-neutral, in a series of seminal works Blau and Kahn brought attention to the importance of the ‘wage structure’ in shaping the gender wage gap. They showed that even if we assume (as they did) that the wage structure is gender-neutral, i.e., the market price for skills is identical for men and women, it can still affect the gender pay gap so long as men’s productivity-related characteristics are superior to those of women. Work experience, one of the main components of human capital, is an excellent example. Women lag behind men in terms of labor market experience, such that even if the rewards for each year of experience are equal for men and for women, men will benefit more than women from work experience simply because they have more of it. As the rewards for experience are higher, so too is the advantage enjoyed by men. Furthermore, if women’s average experience rises (a change at the individual level), the gender wage gap can be expected to narrow. But, if at the same time the wage structure changes such that the returns to experience grow substantially (a change at the structural level), men’s advantage in terms of experience, while shrinking, can still hold the wage gap from decreasing because men benefit more from their remaining advantage (Blau & Kahn, 1997, 2017a).

The effect of the wage structure, according to this logic, is not due to gender differences in rewards to wage-related characteristics (which are assumed to be the same), but due to compositional differences between men and women with regard to wage-related characteristics. Thus, Blau and Kahn, as well as others, have concentrated on the ‘explained’ portion of the gap, i.e., differences berween men and women in key wage-related characteristics such as education and experience. The ‘unexplained’ portion, in contrast, which has been attributed to market failure (discrimination) or measurment error, remained an aggregated unified unit, reflecting the assumption that the wage structure is basically gender-neutral.

The conceptualization of the wage structure as gender-neutral fails to account for systematic differences between the genders in returns to productivity-related characteristics. Because the labor market is embedded in the broader power relations of any given society, we cannot accept the assumption that men and women are subject to the same ‘criteria’ that set workers’ wages; rather, the basic presumption should be that the price that employers are willing to pay for certain productivity-enhancing qualities differ for men and for women. Our empirical motivation for disaggregating this structural component of the gender wage gap is therefore based on our theoretical argument that these ‘criteria’ are gender-specific, rather than gender-neutral. By that, we point at the direct effect of the wage structure, above and beyond its indirect effect via the compositional differences described above. In practical terms, this means that unlike most decomposition analyses, we aim to disaggregate the ‘unexplained’ portion of the wage gap, in order to reveal how gender differences in returns to the two prime components of human capital—education and work experience—affect the gender wage gap.

This is not the first attempt to follow this direction. The idea that wage structures are in fact gender-specific is supported by earlier studies which provide evidence for gender differences in returns to education, work experience, and occupations. These studies differ from ours in their theoretical motivation as well as their empirical analysis. Studies by Dougherty (2005), Goldin (2014), and Munasinghe et al. (2008) examined gender differences in returns to human capital, but did not examine the contribution of these differences to the gender wage gap. In contrast, studies that did examine the contribution of structural aspects to the gender wage gap (e.g., Albrecht et al., 2003; Arulampalam et al., 2007; Chzhen & Mumford, 2011; Filer, 1985) did not look further in order to distinguish between differences in returns to specific characteristics like education and experience, as we do. The rising returns to both education and experience over recent decades (Haelermans & Borghans, 2012; Juhn et al., 1993; Murphy & Topel, 2016) makes the distinction between the two even more crucial for understanding trends in the gender wage gap.

## Decomposition of the Gender Wage Gap

When shifting the focus from gender differences in human capital to gender differences in *returns* to human capital, the standard way of decomposing the gender wage gap has crucial limitations. We propose two deviations from the standard method: put together, these will facilitate accounting for the substantive differences in returns to skills between male and female employees.

There is a long tradition of research using the Oaxaca-Blinder (Blinder, 1973; R. Oaxaca, 1973) decomposition technique to examine how much of the gender wage gap is explained by differences in productive characteristics between men and women (Lips, 2013; Ponthieux & Meurs, 2015). The starting point of this analysis is the Mincerian earnings function, which regresses the log-wage against measures of individual human capital—namely actual work experience and education—and other background variables (Kunze, 2008). The decomposition is based on estimating the earnings function for men and women separately, and then using these estimates in a counterfactual analysis which considers what would happen to the gender wage gap if women had the same characteristics as men. The decrease of the wage gap in such circumstances is considered the ‘explained portion’ of the gap, or the ‘endowments effect’. The residual is attributed to unmeasured variables and discrimination. More formally, the standard decomposition, also known as the twofold decomposition, can be written as:

$${\overline{\mathrm{ln }(W)}}_{m}-{\overline{\mathrm{ln }(W)}}_{f}=\left({\overline{X} }_{m}-{\overline{X} }_{f}\right){\beta }_{m}+\left({\beta }_{m}-{\beta }_{f}\right){\overline{X} }_{f}$$

where W is the hourly wage, X denotes measures of human capital, and β is the returns to human capital estimated by the male and female wage equations, labelled *m* and *f* respectively.

There are two problems with this widely used formulation. The first problem arises from the implications of using semi-log models in which the logarithms of wages are estimated. The coefficients in a semi-log model do not represent the absolute marginal effect of the covariates on the dependent variable, but rather the relative effect (i.e., the percentage change in the dependent variable due to an absolute change in the covariates). In the case of the earnings function, this means that the coefficients serve as estimates of the *rate of return to investment in human capital,* and do not represent the monetary gain with each unit increase in the covariates. The rate of return fits the neoclassical economic perspective, which conceives human capital in terms of an investment made by individuals, and the benefits that accompany human capital as returns to investment. According to this perspective, the rational decision regarding whether to invest in education, for example, depends on the expected benefit, given your gender and your expected earnings otherwise.

Because the coefficients in a semi-log model represent the percentage—rather than absolute—change in the dependent variable, the coefficients are not comparable across gender groups. The relative returns estimated in the male equation are relative to the male distribution of wages, while those in the female equation are relative to the female distribution. Because the baselines are different for the two gender distributions, a 10% wage increase in the women’s distribution is worth less (in absolute terms) than a 10% increase in the men’s distribution. To give a concrete example, Diprete and Buchmann (2006) used CPS data to estimate returns to education by comparing the earnings of college graduates and high school graduates. They found that in 2000, returns to college education was higher for women than for men; white women could expect a 118% increase in their earnings (relative to high-school graduate women), as compared to only 69% increase for men. However, this higher college premium is actually worth less than men’s in absolute terms: it translates to $10,959 premium, as opposed to $12,353 for men. Thus, comparisons of the relative returns to a specific skill provide important information on the benefit of that skill for men and women given their potential earnings otherwise; but little information on gender inequality in returns to that specific skill.

The unchallenged convention of always using the log-wage in statistical models, together with the predominant conceptualization by economists of human capital as an individual’s investment, has led to the misrepresentation of the gender gap in returns to education and experience—even in studies that were focused on precisely this. The comparison of rates of return between men and women can tell us which gender group has a stronger incentive to invest in acquiring human capital given its potential earnings otherwise; but it does not inform us about gender differences in the real value of these investments (Pekkarinen, 2012). Since unskilled women tend to earn much lower wages than unskilled men, the relative returns of each gender are calculated from very different baselines. Indeed, the findings of Diprete and Buchmann (2006) cited above show that the within-group relative returns to college education were higher for women than for men; but this does not mean that men get less for their education. Even if the relative returns to skills were exactly the same—a state of affairs that can be defined as zero-discrimination conditions—the absolute wage increase associated with these skills may still be substantially different. What should be compared, then, are the absolute returns.

The second problem with the standard decomposition arises from the fact that it adopts the coefficients from the male earnings function as representative of the wage structure. Thus, the counterfactual scenario that is being tested is one in which men and women have equal skills *and* are rewarded for them as men currently are. This issue has long been recognized, and revisions to this basic application use ‘middle-ground’ coefficients representing a nondiscriminatory wage structure (R. L. Cotton, 1988; Neumark, 1988; Oaxaca & Ransom, 1994). Either way, the choice of male coefficients, female coefficients, or midpoint coefficients as the reference point assumes a uniform gender-neutral wage structure, which cannot account for the possibility that rewards to human capital are gender-specific. In other words, it does not allow for estimating the contribution of gender-specific wage structures to the gender wage gap. Furthermore, a decomposition that relies on a standard monolithic wage structure (by using the men's, women's, or midpoint coefficients) contaminates the ‘explained’ portion of the wage gap with the deviations from this standard. Part of the ‘explained’ portion is not due to differences in skills alone, but actually due to differences in coefficients (Elder et al., 2010). For example, if the male coefficients are used as the standard (as in the equation above), the ‘explained’ portion of the gap consists of the part of the gap that is due to differences in human capital between men and women *if women had men’s coefficients* – which they most likely do not. This method, thus, overestimates the ‘explained’ portion of the wage gap, and downplays the contribution of the structural component, i.e., the differences in returns to human capital.

What we propose as a solution to the problems raised above is, first, to examine the gender wage gap in absolute—rather than relative—terms; and second, to use a threefold—rather than a twofold—decomposition. As noted, the use of the absolute wage as the dependent variable renders the coefficients comparable between men and women, as they are not dependent on men’s and women’s different baselines (the constants in the male and female wage equations). Therefore, the gap between men’s and women's coefficients represents gender inequality in the value of labor market skills. The threefold decomposition (Jann, 2008) allows isolating the contribution of differences in the covariates and the contribution of differences in the coefficients of each covariate to the overall wage gap. It divides the wage gap into three parts: (1) the endowments effect, i.e., the part explained by differences in men’s and women’s characteristics, but this time without changing the returns that women currently receive; (2) the 'wage structure' i.e., the part explained by differences in returns/market prices to these characteristics; and (3) the interaction between differences in endowments and coefficients. Formally, it can be written as:

$${\overline{W} }_{m}-{\overline{W} }_{f}=\left({\overline{X} }_{m}-{\overline{X} }_{f}\right){\beta }_{f}+\left({\beta }_{m}-{\beta }_{f}\right){\overline{X} }_{f}+\left({\overline{X} }_{m}-{\overline{X} }_{f}\right)\left({\beta }_{m}-{\beta }_{f}\right)$$

This threefold decomposition allows assessing separately what would happen to the wage gap if women had the same characteristics as men but kept their current returns; and what would happen if women were rewarded for their current characteristics equally to men. Since gender differences in characteristics have declined over time, we can expect that the role of these differences in explaining the wage gap should decline as well.

## Analysis and Data

Our analysis and data are motivated from and based on a recent publication by Francine Blau and Lawrence Kahn (Blau & Kahn, 2017a), which provides an excellent and comprehensive summary of the research, and an updated empirical account of the gender wage gap in the United States. As discussed above, we employ two modifications to the standard decomposition method used by Blau and Kahn (2017a): estimating the absolute, rather than relative, effects of human capital; and using a threefold, rather than twofold, decomposition. These modifications allow us to present an estimate of the real economic rewards to human capital received by men and women, and then to estimate the part of the wage gap that can be attributed to the differences in these rewards. Unlike previous studies, we distinguish between the rewards for education and work experience, in order to better understand the effect of the wage structure on the gender wage gap. Lastly, we conduct this analysis at two points in time, in order to capture changes in the role of the gender-specific wage structure over time.

### Data and Variables

We analyze the gender wage gap at two time points, 1980 and 2010. Blau and Kahn’s analysis and findings serve as the reference point for our empirical analysis. For consistency, we use the same data, same variables, and same specification as in their human capital specification model (ibid, Table 4, panel A), in which the hourly wage is regressed on education and work experience, controlling for race and region.^1^ Blau and Kahn used data from the Michigan Panel Study of Income Dynamics (PSID), which is the only dataset available with detailed information on the actual work experience^2^ of respondents from all cohorts. We use the same dataset, which they have made available online (Blau & Kahn, 2017b). Following Blau and Kahn, we focus on working-age (25–64) non-farm employees who worked full-time for at least 26 weeks during the year preceding the survey. This focus allows comparing men and women with similar levels of commitment to the labor market.

The dependent variable in the analysis is the inflation-adjusted hourly wage. The independent variables include the following: 1) *Gender*; 2) *Education—*measured by two variables, years of schooling and academic degree (in three categories: no degree, BA, and postgraduate degree); 3) *Work experience—*we follow Blau and Kahn and others in differentiating between experience in full-time work, which is associated with positive returns, and experience in part-time work, which is indicative of low earnings and hence negative returns (Gornick & Jacobs, 1996; Olsen & Walby, 2004). The fact that the PSID data include information on the work history of each respondent is what puts them in a unique position to provide the most accurate measures of actual work experience, as opposed to the frequently used estimated experience based on age and years of schooling (Blau & Kahn, 2013); 4) Race—in four categories: white, black, Hispanic, and other; 5) Region – in four categories; and 6) residence in metropolitan vs. rural areas.^3^

## Findings

**Fig. 1:**
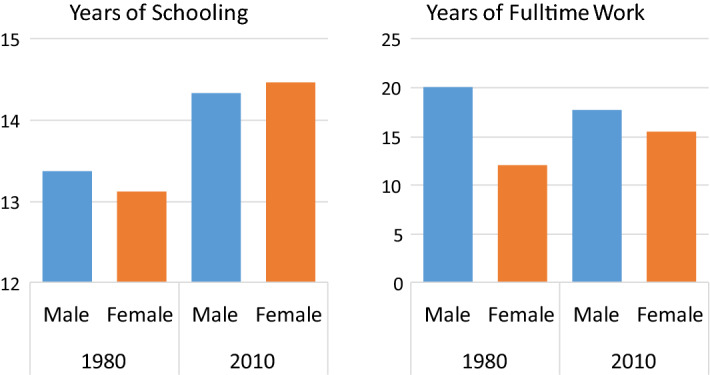
Gender differences in mean years of schooling and full-time work experience

Figure 1 displays a narrowing of the gender gap in the two prime components of human capital, average years of schooling and full-time work experience. In 1980, women had a slightly lower average number of years of schooling compared to men (13.12 versus 13.37), but by 2010, the balance had been reversed (14.47 for women versus 14.33 for men). The difference in years of full-time work was much larger in 1980 (12.09 for women versus 20.05 for men) before narrowing substantially by 2010 (15.54 versus 17.68 for women and men, respectively). As discussed at the outset, gender differences in education and work experience constitute the ‘explained’ portion of the gender wage gap. As these differences converge over time, we can expect their explanatory power to decline.

Blau and Kahn report that the women-to-men earnings ratio increased from 62% in 1980 to 79% in 2010. They followed the tradition of decomposing the log-wage differentials using the male coefficients, finding that differences in human capital between men and women explained 26.6% of the wage gap in 1980 and only 8% in 2010.^4^ This should come as no surprise, given the growing educational attainments of women and the increase in the extent of their work experience. In contrast, the ‘unexplained’ portion of the gap grew from 71.4% in 1980 to 85.2% in 2010. Whilst Blau and Kahn acknowledge that the unexplained portion is the main source of gender inequality, not much of the discussion is dedicated to these differences in returns, and the contribution of specific elements of these differences, namely education and experience, are not reported. Nonetheless, these findings are important because they highlight the relatively small and declining importance of gender differences in human capital in explaining women’s lower wages, rebutting the claim that women’s wage disadvantage reflects their lower skills.

**Fig. 2:**
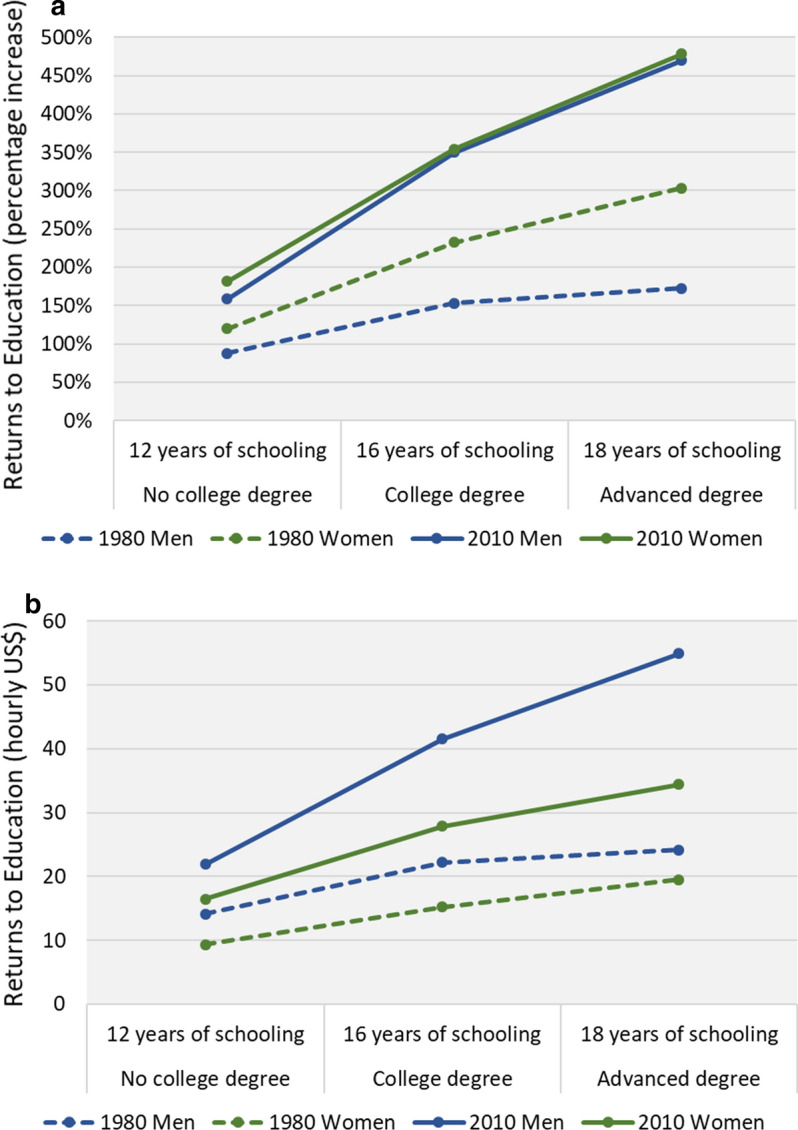
**a** Relative returns to education. **b** Absolute returns to education

**Fig. 3:**
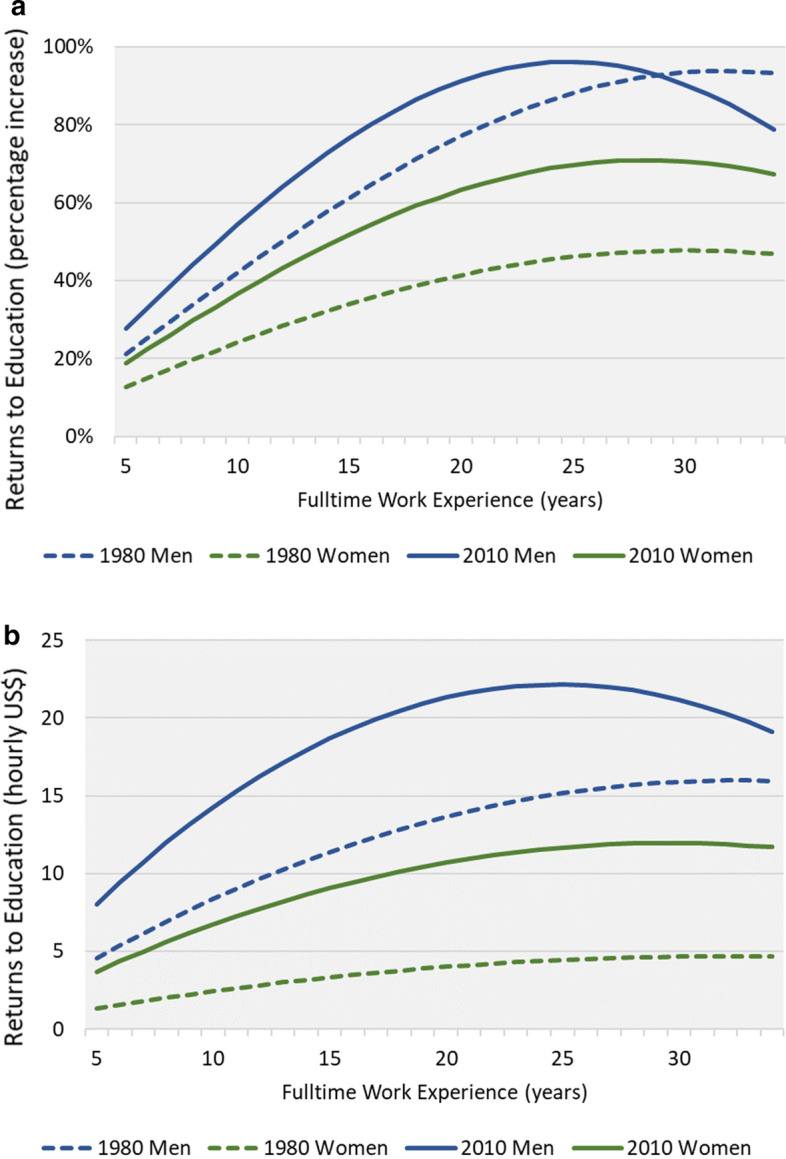
**a** Relative returns to full-time work experience. **b** Absolute returns to full-time work experience

As discussed above, we have introduced two changes to Blau and Kahn’s analysis: we use the real hourly wage instead of its log transformation, and employ a threefold decomposition instead of its regular twofold form. We also differ from Blau and Kahn in our basic assumption that returns to human capital are gender specific; thus, our first step is to test whether differences in returns between men and women do exist. To this end, we begin by presenting findings from an OLS regression of hourly wages where education and experience are both interacted with gender, to examine whether and to what extent men’s and women’s coefficients differ. Note that although we focus on education and experience, the model also controls for Region and Race, replicating Blau and Kahn's human capital model. We conducted this analysis in two versions: using the log-wage in one and the real (inflation-adjusted) wage in the other, to concretize the difference between the rates of return (derived from the semi-log model) and the absolute returns (derived from the real wage model). The results of these analyses are presented in Table 1, and the more substantial findings are outlined in Figs. 2 and 3.

**Table 1:** OLS regression of log- and real-hourly wage

	Log-Wage Models						Real-Wage Models			
	1980			2010			1980		2010	
	b	s.e	exp(b)	b	s.e	exp(b)	b	s.e	b	s.e
Female	− .39**	(.12)	0.67	− .17	(.18)	0.84	5.24	(2.81)	10.08	(7.89)
Full-time exp	.04**	(.00)	1.04	.05**	(.00)	1.06	.99**	(.08)	1.79**	(.18)
Full-time exp.*Female	− .02**	(.01)	0.98	− .02**	(.01)	0.98	− .70**	(.13)	− .98**	(.26)
Full-time exp. sq	− .0007**	(.00)	1.00	− .0011**	(.00)	1.00	− .015**	(.00)	− .036**	(.00)
Full-time exp. sq.*Female	.0002	(.00)	1.00	.0004**	(.00)	1.00	.011**	(.00)	.022**	(.01)
Part-time exp	− .01	(.01)	0.99	− .01	(.01)	0.99	− .14	(.16)	− .08	(.35)
Part-time exp.*Female	− .01	(.01)	0.99	.00	(.01)	1.00	.11	(.20)	− .06	(.41)
Part-time exp. sq	.00	(.00)	1.00	.00	(.00)	1.00	.00	(.01)	− .02	(.03)
Part-time exp. sq.*Female	.00*	(.00)	1.00	.00	(.00)	1.00	.01	(.01)	.03	(.03)
Years of schooling	.05**	(.00)	1.05	.08**	(.01)	1.08	1.18**	(.12)	1.82**	(.39)
Years of schooling*Female	.01	(.01)	1.01	.01	(.01)	1.01	− .40*	(.20)	− .46	(.58)
B.A	.09**	(.03)	1.10	.24**	(.04)	1.27	3.42**	(.74)	12.32**	(1.65)
M.A	.06	(.04)	1.06	.32**	(.06)	1.37	3.01**	(1.00)	22.04**	(2.43)
B.A.*Female	.06	(.05)	1.06	− .10	(.06)	0.90	− .68	(1.24)	− 6.35**	(2.41)
M.A.*Female	.15*	(.07)	1.16	− .11	(.08)	0.89	2.53	(1.71)	− 12.25	(3.51)
Black	− .11**	(.02)	0.89	− .21**	(.02)	0.81	− 2.33**	(.53)	− 5.03**	(.97)
Hispanic	− .12**	(.04)	0.89	− .01	(.04)	0.99	− 1.12	(.98)	.19	(1.52)
Other race	− .01	(.09)	0.99	− .03	(.06)	0.97	− .41	(2.22)	− .28	(2.68)
msa	.17**	(.01)	1.19	.09**	(.02)	1.10	3.21**	(.36)	2.06**	(.66)
Region: North-east	.00	(.02)	1.00	.07	(.02)	1.07	− .09	(.51)	2.86	(1.02)
Region: North-central	.01	(.02)	1.01	− .10**	(.02)	0.90	− .21	(.49)	− 3.37**	(.94)
Region: South	− .08**	(.02)	0.92	− .09**	(.02)	0.92	− 1.42**	(.49)	− 2.70**	(.94)
Constant	1.78**	(.08)	5.94	1.52**	(.13)	4.59	− 5.62**	(1.81)	− 16.19**	(5.43)
R^2^	0.43			0.33			0.34		0.21	
N	4,276			5,474			4,276		5,474	

*p < .05, ** *p* < .01

### Gender Differences in Returns to Skills

Figure 2A illustrates the effect of education (based on both degree and years of schooling) on wages, by gender and year. Note that in Fig. 2a, returns are expressed as a percentage increase, as they are based on the semi-log models. The figure shows that between 1980 and 2010 both men’s and women’s lines moved upwards, reflecting the overall increase in returns to education. The figure suggests that women had higher relative returns to education in 1980 but had lost this advantage by 2010. Indeed, the first column of Table 1 displays positive interaction terms between education and gender, suggesting that in 1980 women (coded as 1) tended to have higher rates of return to their education. But this gender gap in returns to both BA education and years of schooling is only on the brink of statistical significance,^5^ and it is only the interaction with postgraduate education that manifests statistical significance.^6^ The combined effect of years of schooling *and* degree attainment, as presented in Fig. 2a, can be comprehended more intuitively. The figure shows that in 1980 possessing a postgraduate degree and 18 years of schooling added 172% to the earnings of men (compared to men with no schooling) and 303% to the earnings of women. However, as Fig. 2a clearly shows, these differences in returns had dramatically narrowed by 2010; possessing a postgraduate degree and 18 years of schooling added around 475% to the earnings of *both* men and women.

When the dependent variable is the absolute wage (in US$), a different picture emerges. The third and fourth columns of Table 1 present the results of the real wage models for 1980 and 2010 respectively, and Fig. 2b illustrates the combined absolute effect of education on wages by gender and year. Here too, the overall increase in returns to education between 1980 and 2010 is evident. More importantly, the figure shows that in both years, women had *lower* absolute returns to their education, and that the gap increased considerably between 1980 and 2010, especially in the case of higher education. The marginal effects reported in Table 1 suggest that the hourly wage premium for a bachelor’s degree in 2010 was $12.3 for men, compared to only $6 for women. For advanced degrees, the gap increased further, with a $22 premium for men and $9.8 for women. At both levels, then, the premium for higher education was more than double for men – an important finding which is completely obscured when returns to education are expressed in relative terms. The contrast between Fig. 2a and b is striking. Although both show that returns to education are gender specific, the latter shows a clear advantage for men, in all education categories and for both time-periods, which cannot be seen in the former, where education premiums are presented as rates of return.

Turning to experience, it is important to note that due to the nonlinear effect of experience (each year of experience has a stronger effect during the earlier stages of one’s career) the models include a quadric term of experience. As a consequence, it is harder to interpret the results from the table, especially since all the experience variables are also interacted with gender. To clearly demonstrate the effect of experience in both models, we plotted the wage premiums across the scale of full-time experience and we relate to the graphic representation. Figure 3a presents the rates of return (as a percentage) and Fig. 3b presents the absolute hourly wage premium, based on the log-wage and real wage models respectively. The two figures show, first, that the positive effect of experience on earnings is indeed nonlinear, as it weakens as we move towards the higher end of the scale. Second, the figures show that the increase and then decrease in returns at the beginning and at the end of a lifetime career (lower and then higher values of experience) are sharper among men in 2010. Third, between 1980 and 2010, both men’s and women’s lines moved upwards, reflecting the overall increase in returns to experience. Lastly, and most importantly for the current discussion, the figures show that in both years, men benefited from their experience substantially more than women, and the findings are similar whether it is relative or absolute returns being considered. Nevertheless, in the case of relative returns, the gender gap appears to have declined between 1980 and 2010, whereas the gender gap in absolute returns remained at a similar level.

The findings presented so far support our arguments in two ways. First, they illustrate the substantive difference between the rates of return estimated by the semi-log model and the absolute returns estimated by the real wage model, and show that they are not equivalent. To reiterate, the relative returns give us the percentage increase in wages due to education and work experience, based on each group’s distinctive distribution, and thus are informative for learning about the different incentives men and women have for investing in their human capital. They are not, however, useful for comparisons between groups. Second, the findings show that men and women do not receive the same returns to their productive skills. Women’s absolute returns to both education and experience are substantially lower than men’s, meaning that the market rewards to human capital are not gender-neutral.

### Decomposing the Real Hourly Gender Wage Gap

**Fig. 4:**
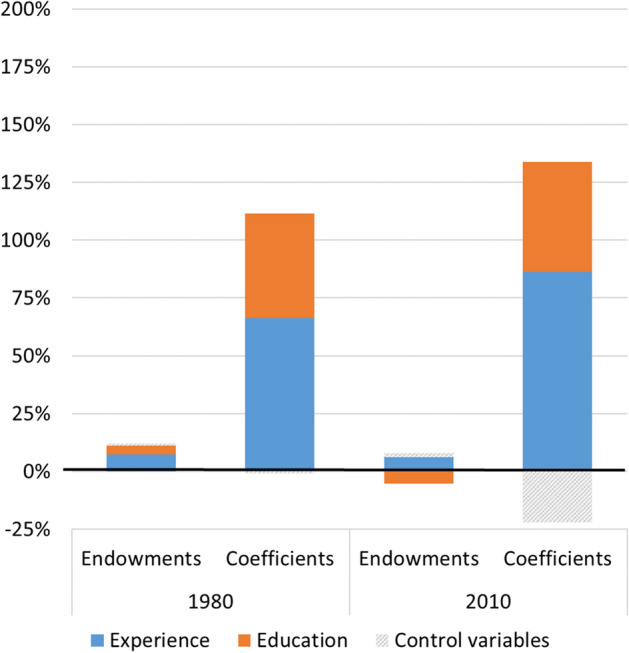
Blinder-Oaxaca threefold decomposition of the gender wage gap (selected findings)

These findings lead to the next step of our analysis: examining the contribution of gender differences in levels of education and experience ("explained"), versus the contribution of gender differences in returns to these skills ("unexplained"), to the gender wage gap. We do so by decomposing the real hourly wage gap between men and women in 1980 and 2010. Table 2 reports the results, and Fig. 4 illustrates the key findings from this analysis. Since our theoretical interest here is in a comparison of returns by gender, we only decompose the gender gaps in absolute wages.

**Table 2:** Blinder-Oaxaca threefold decomposition of the gender wage gap

		1980	2010
Real hourly wage	Male	24.2	31.8
	Female	15.0	23.7
	Difference	9.2	8.1
Endowments	Experience	7.3%	6.3%
	Education	3.7%	− 5.2%
	Control variables	1.1%	1.7%
Coefficients	Experience	66.2%	86.4%
	Education	45.4%	47.6%
	Control variables	− 0.8%	− 22.0%
Constant		− 45.8%	− 22.8%
Interactions		22.9%	8.1%
Total		100%	100%

As shown in Table 2, the estimated gap between men’s and women’s hourly wages was $9.2 in 1980, declining to $8.06 by 2010. As a threefold decomposition was used, the ‘explained’ portion of the gap represents only the differences in characteristics between men and women, and is isolated from the gender differences in returns. As expected, the contribution of the former to the wage gap is small: 12.1% in 1980, and only 2.7% in 2010. This small effect indicates how important it is to pay much more attention to the ‘unexplained’ portion of the gap. More specifically, in 1980 gender differences in educational attainments explained 3.7% of the wage gap. In 2010 women’s level of education was already higher than men’s. Therefore, education had a suppressing effect on the wage gap; equalizing women’s education level to that of men’s would have *increased* the wage gap by 5.2%. In both years, then, education differences either had a small effect on the wage gap or obscured it.

The results concerning experience are similar. Women, on average, have less experience than men; while the gap shrunk over time, they still lag behind (see Fig. 1). Accordingly, this difference explained 7.3% of the wage gap in 1980, and even less—6.3%—in 2010. These figures are smaller than those reported by Blau and Kahn (23.9% and 15.9% in 1980 and 2010, respectively), indicating that the standard twofold decomposition contaminates the 'explained' effect with the effect of the returns differential; while simultaneously failing to correctly account for the substantive differences between men’s and women’s returns, due to its reliance on log-wage equations. In a threefold decomposition of the log-wage gap (not presented here), distinguishing between the effect of different characteristics and different rates of return, we found that the portion explained by experience was 13.3% and 9.2% in 1980 and 2010, respectively. These figures are much closer to the findings from the decomposition of the real wage gap than to Blau and Kahn’s original (log-wage) findings—pointing again at the necessity of isolating the effect of the market returns to skills (i.e., the gendered wage structure) from the effect of differences in human capital.

In contrast to the effect of differences in human capital, then, differences in returns to human capital played a much larger and increasing role in explaining the gender gap, as Fig. 4 clearly shows. If women had the same returns to education as men, the wage gap would have narrowed by 45.4% in 1980 and 47.6% in 2010. An even larger portion of the wage gap is attributed to differences in returns to experience. Specifically, if women had the same returns to experience as men, the wage gap would have narrowed by 66.2% in 1980, and 86.4% in 2010. As gender differences in human capital are diminishing while gender differences in returns persist or even increase, it is unsurprising to find that the gender wage gap is largely shaped by the latter.

Two interesting points arise from the findings, pointing at the interdependence of the factors that are at play. With regard to work experience, the portion of the gender wage gap that is due to differences in returns to experience increased substantially, even though gender differences in these returns were similar in 1980 and 2010 (see Fig. 3b). This conundrum can be resolved if we take into account the fact that returns to experience rose considerably between the two periods, and therefore had much greater weight in 2010, even if the differences in returns remained roughly the same. As for education, returns to education for both genders increased between 1980 and 2010; at the same time, differences between men and women in returns to education expanded (see Fig. 2b). These trends could lead us to expect that the portion of the wage gap explained by differences in returns to education would increase over time, but the findings show otherwise. This is possibly because the advantage gained by women in terms of acquiring higher education was enough to offset the effect of their growing disadvantage in terms of returns to education.

To provide further validation to the conclusion above we apply the same decomposition after disaggregating the analysis by two broad categories of occupations and industries. Specifically, we distinguish between manual and semiprofessional occupations vs. professional and managerial occupations, and between the manufacturing industry versus the service industry. The results (presented in the appendix) show that in all four segments most of the gender wage gap is related to gender differences in returns (i.e., the wage structure/coefficients), and only a small fraction is related to gender differences in skills between men and women (endowments). That said, there is a clear variation between segments in the extent to which gender differences in returns to skills explain gender differences in pay. Understanding this variation, though, would require delving into the unique characteristics of each segment, which is beyond the scope of this paper. We therefore refer here to the most significant segment with regard to overtime changes in gender gaps in recent decades—the managerial and professional occupations.

Managerial and professional occupations not only have the highest levels of education and skills and offer greater rewards for skills, but also found to be specifically important in relation to women’s upward mobility in the labor market, and the gender pay gaps in particular (Hegewisch & Hartmann, 2014). The rise of women’s education and skills in the past decades has motivated women’s entry into highly paid male-dominated occupations. This has led to one of the most important changes in gender inequality in the labor market: the decline of gender occupational segregation (England, 2010). Of a particular importance is the inflow of women into high paying managerial and professional occupations (Mandel, 2012; Roos & Stevens, 2018). Between 1980 and 2010 women increased their share in these occupations from 26% to 43% (authors’ calculations from the Current Population Survey data (US-CPS)). While in 1980 only 9% of women worked in these occupations, in 2010 this figure nearly doubled, reaching 17.1% (as compared to 21.5% of men). This trend may seem as a promising path to greater gender equality, but the reality is more complicated. Wages in these occupations are higher, but so is wage inequality (Gottlieb et al., 2019), which reflects on gender inequality. While the overall gender pay gap has declined between 1980 and 2010, it actually has not changed within the managerial and professional occupations, as the women to men wage gap remained at about 69%.

**Fig. 5:**
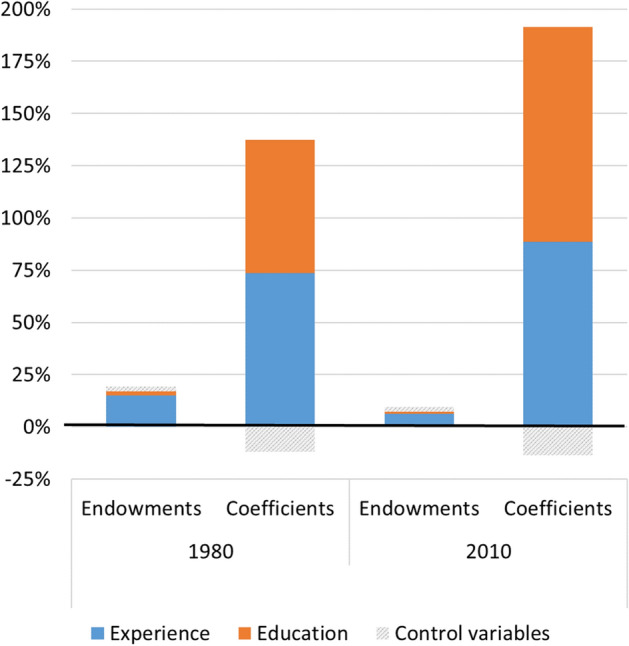
Blinder-Oaxaca threefold decomposition of the gender wage gap among employees in managerial and professional occupations (selected findings)

Table 3 and Fig. 5, which present the results of the decomposition analysis after limiting the sample to workers in managerial and professional occupations, reveal similar but more pronounced results as those found in the entire labor market. Specifically, Fig. 5 shows that, as found earlier, the portion of the gap explained by gender differences in skills is small and in decline (falling from 19.1% in 1980 to 9.6% in 2010). In contrast, the portion of the gap explained by gender differences in returns to skills is much larger. Furthermore, between 1980 and 2010 the portion of the gap explained by gender differences in both experience and education substantially increased. The portion related to returns to education as well as the portion related to returns to experience are much larger in these occupations. These results indicate that in the more lucrative jobs the unequal returns to skills serve as an even greater obstacle for achieving gender equality. Given women’s rapid integration in these occupations, and the opportunity they open for women’s economic advancement, the restrictions that the wage structure imposes on women’s relative wage become more evident.

**Table 3:** Blinder-Oaxaca threefold decomposition of the gender wage gap among employees in managerial and professional occupations

		1980	2010
Real hourly wage	Male	28.9	42.8
	Female	19.8	29.8
	Difference	9.1	13.0
Endowments	Experience	14.9%	6.4%
	Education	2.0%	0.7%
	Control variables	2.2%	2.5%
Coefficients	Experience	73.5%	88.7%
	Education	63.8%	102.9%
	Control variables	− 12.0%	− 13.5%
Constant		− 80.4%	− 93.0%
Interactions		36.0%	5.4%
Total		100%	100%

It is important to highlight that the current analysis, which follows Blau and Kahn’s (2017a) work, focuses on workers that demonstrate relatively high commitment to the labor market (working full-time and for at least 26 weeks in the year before they were surveyed). Within this group, we found that human capital differences have little effect on the gender wage gap. But they may still have a larger effect on the gender gap outside the primary labor market, where there are more women than men, and where levels of human capital, as well as wages, are lower. Thus, our analysis does not capture the total gender wage gap in the labor market. In fact, our evaluations of the wage gap are likely to be underestimated, given that the women in our sample are more selective than the men. Our findings thus show that it is within the primary labor market—exactly where human capital is rewarded the most and where high-skilled women can expect to realize their earnings potential—that women find themselves subject to a different set of rules compared to men. This is what we term as a gender-specific wage structure, and it begs the obvious question: why is it so? We propose a few possible answers to this question in the concluding section of this paper.

## Discussion

This paper seeks to fill a theoretical and empirical lacuna in the current literature on gender wage inequality in the labor market. Theoretically, the paper challenges predominant conceptualization of the wage structure as gender-neutral, rather emphasizing the contribution that it makes to the gender wage gap. Considering previous empirical evidence, most studies that decomposed the gender wage gap dedicated much of their attention to that part of the gap explained by gender differences in productivity-enhancing characteristics (i.e., the ‘explained’ portion). The ‘unexplained’ portion of the gap, which refers to the residual that cannot be explained by differences in characteristics, stems from the wage structure, i.e., the market returns to human capital. These returns have been assumed to be gender-neutral, and their relation to gender mediated by differences in human capital between the gender groups. Gender differences in these returns were attributed to market failure (discrimination) or measurement error, and thus received little scholarly attention in decomposition research. Studies that did document gender differences in returns to human capital usually did not examine the contribution these differences made to the gender wage gap. Furthermore, these studies focused on the rates of return to investment, i.e., gender differences in the *relative* (gender specific) value of human capital, neglecting the differences between men and women in the *absolute* monetary value of human capital—the measure that matters when addressing gender inequality.

Our findings show that the key to understand the gender wage gap and its persistence lies not in the different characteristics of male and female workers, but mainly in the fact that women are rewarded less than men for their skills. Gender differences in absolute returns to human capital are prevalent and account for much of the wage gap, and their effect increases over time. This conclusion holds even when disaggregating the labor market to broad categories of occupations and industries. Further research is needed to identify the sources of the gender differences in returns; the first step, made by the current paper, is to acknowledge their importance to the remaining gender pay gap.

In search for explanations for differential returns to human capital, we need to distinguish between two types of explanations: those that rely on differences in the behaviors of men and women in the labor market, and those that focus on the structure of the labor market. The first type relates to identifying the mechanisms and processes that explain how and why women, as individuals, end up in positions with lower rewards. Socialization and social control explain women’s compliance with norms that channel them into positions and employment patterns that pay less. This includes, first and foremost, the consequences of women’s commitment to unpaid domestic and care work, especially childrearing, that hinders their potential to obtain pay raises and promotions—or, in other words, to maximize the rewards for their skills (Budig & England, 2001; England et al., 2016). One example is the fact that women’s availability for working extra hours is constrained by their commitment to domestic and care work, and so they are less likely to exploit the wage premium enjoyed by workers who can work long hours and overtime (Cha & Weeden, 2014). Gender norms and expectations at early life stages also account for segregation between qualitative categories within quantitative educational levels. The outcome is that women are directed into fields that pay less due to factors such as reduced workload or low productivity. These mechanisms, which operate at the individual level, not only account for the different human capital acquired by men and women (the explained portion) but also for the lower rewards that women get for the “same” human capital (unexplained), or the gender pay gap within the same occupation.

The second type of explanations for differential returns to human capital consists of explanations that focus on the structural mechanisms and processes that shape the different opportunity structures that men and women face in the labor market. At the macro level, deeply rooted gender beliefs lead to the low evaluation of fields, jobs and tasks predominantly undertaken by women or signified as feminine. In this case, the lower rewards to a job or occupation apply to all workers, men as well as women. Nevertheless, since women by definition tend to work in female-dominated jobs and occupations, the outcome is that many women receive low rewards for their skills because they acquired female-typical skills that are undervalued by the labor market. Furthermore, as more women enter fields and occupations that were traditionally dominated by men, these fields and occupations suffer from devaluation and the rewards they offer decline (England, 1992; Levanon et al., 2009; Mandel, 2018). Note that gender inequality also exists within occupations, partly due to the lower evaluation of specific tasks—predominantly those carried out by women (Bizopoulou, 2019; Goldin, 2014). Supporting this claim, when focusing on the managerial and professional occupations, high paying occupations to which women have entered in masses in the past few decades, we indeed find that in these occupations a greater portion of the gap is related to differences in the returns to skills between men and women (especially to education), and that this portion increases even further over time.

On a final note, the decomposition of the gender wage gap into ‘explained’ and ‘unexplained’ portions, and its identification with ‘individual’ and ‘structural’ mechanisms of gender inequality is not merely analytical but has particular importance for policymaking. It points at the elements that have the largest impact on the gender wage gap, and therefore can guide the design of interventions that would be most effective and meaningful. Normative changes that encourage women (and girls) to invest in their human capital have certainly achieved a lot; women have made substantial advancements in the acquisition of labor market skills. However, the results presented in this paper indicate that it is vital to focus more on the institutional and structural factors that prevent women from fully utilizing their skills. Policymakers who wish to target the gender wage gap should therefore be aware of the economic consequences of socialization and gender beliefs that account for the low evaluation of women, as well as their education, skills, and occupations.

## Appendix: Decomposition of the gender wage gap – disaggregation by categories of occupations and industries

**Table:**

		Semiprofessional & manual occupations		Managerial & professional occupations		Manufacturing industry		Service industry	
		1980	2010	1980	2010	1980	2010	1980	2010
	Male	21.2	24.4	28.9	42.8	25.0	31.3	23.6	32.0
	Female	12.8	18.2	19.8	29.8	13.5	25.3	15.4	23.5
	Difference	8.4	6.2	9.1	13.0	11.5	6.0	8.1	8.4
Endowments	Experience	7.5%	6.2%	14.9%	6.4%	5.7%	10.8%	7.7%	4.5%
	Education	− 2.1%	3.5%	2.0%	0.7%	11.1%	− 11.0%	8.2%	1.2%
	Control variables	0.5%	1.7%	2.2%	2.5%	2.5%	− 0.6%	1.2%	3.3%
Coefficients	Experience	59.5%	59.8%	73.5%	88.7%	58.7%	− 53.1%	72.9%	87.5%
	Education	− 41.3%	22.4%	63.8%	102.9%	49.1%	188.1%	82.5%	36.2%
	Control variables	22.0%	− 25.5%	− 12.0%	− 13.5%	18.6%	− 21.0%	− 7.5%	− 18.8%
Constant		41.0%	12.1%	− 80.4%	− 93.0%	− 67.3%	− 65.7%	− 85.0%	− 23.3%
Interactions		27.0%	19.9%	36.0%	5.4%	21.6%	52.3%	19.9%	9.3%
Total		100%	100%	100%	100%	100%	100%	100%	100%

## Footnotes

1 Blau and Kahn also present a more inclusive specification, that controls for categories of occupations and industries (fourteen industry and twenty occupation dummy variables are included in the model). However, interpreting the returns to skill while controlling for segments that has almost no variation in education is questionable. We avoided including the occupations and industry variables, not only because their interpretation is ambiguous (as Blau and Kahn correctly acknowledge (p. 797)), but also because they are inherently related to education and experience. Thus, controlling for them would mask the gender differences in these returns (Reid and Rubin 2003). We do, however, disaggregate the results by broad categories of occupations and industries. The findings are presented in the appendix and discussed in the findings section.

2 Instead of the common, and problematic, measure that approximates work experience by the subtraction of the number of years of schooling plus six from the respondent’s age (Blau and Kahn 2013; Olsen and Walby 2004).

3 See the appendix in Blau and Kahn’s (2017a) paper for more information about the construction of the variables.

4 More specifically, the part of the wage gap explained by education differences was 2.7% in 1980 and -7.9% in 2010 (suggesting that equalization of education in that year would *increase* the wage gap, as women attained more education than men despite earning less); the part explained by differences in work experience was 23.9% and 15.9% in 1980 and 2010 respectively.

5 Note that in a model that includes years of schooling as a single measure of education, we do find that in 1980 (but not in 2010) there is a statistically significant interaction with gender, consistent with previous studies that found that women’s rates of return to schooling are higher than men’s during the 1980s and 1990s (Dougherty 2005; Hubbard 2011).

6 When calculating the net effect of postgraduate education (controlling for years of schooling), we find that in 1980, a postgraduate degree led to a 23% wage premium on average for women, as compared to 6% for men.
